# Hematopoietic cell transplantation using treosulfan-based conditioning for IL-10 receptor deficiency

**DOI:** 10.70962/jhi.20260123

**Published:** 2026-10-15

**Authors:** Madhavi Lakkaraja, Erica G. Schmitt, Amika K. Sood, Karin Chen, Arunkumar J. Modi, James W. Verbsky, Troy Torgerson, Ghassan Wahbeh, Aleksandra Petrovic, K. Scott Baker, Megan A. Cooper, Lauri M. Burroughs

**Affiliations:** 1 Clinical Research Division, Fred Hutchinson Cancer Center, Seattle, WA, USA; 2 Department of Pediatrics, Division of Hematology-Oncology/BMT, University of Washington School of Medicine, Seattle, WA, USA; 3 Department of Pediatrics, Division of Rheumatology and Immunology, Washington University School of Medicine, St. Louis, MO, USA; 4 Department of Pathology and Immunology, Washington University School of Medicine, St. Louis, MO, USA; 5 Department of Medicine, Division of Allergy/Immunology, The University of North Carolina at Chapel Hill, Chapel Hill, NC, USA; 6 Department of Pediatrics, Division of Immunology, University of Washington School of Medicine, and Seattle Children’s Research Institute, Seattle, WA, USA; 7 Division of Bone Marrow Transplant and Cellular Therapies, Arkansas Children’s Hospital, University of Arkansas for Medical Sciences, Little Rock, AR, USA; 8 Department of Pediatrics, Division of Rheumatology, Medical College of Wisconsin, Milwaukee, WI, USA; 9 Allen Institute for Immunology, Seattle, WA, USA; 10 Department of Pediatrics, University of Washington School of Medicine, Seattle, WA, USA; 11 Department of Pediatrics, Division of Gastroenterology, University of Washington School of Medicine, Seattle, WA, USA

## Abstract

IL-10 receptor deficiency is a life-threatening inborn error of immunity associated with early-onset inflammatory bowel disease. Hematopoietic cell transplantation (HCT) using treosulfan-based conditioning resulted in excellent survival with full donor engraftment. Importantly, anakinra effectively served as a bridge to HCT.

## Teaser

Lakkaraja and Schmitt et al. observe that IL-10 receptor deficiency is a life-threatening inborn error of immunity associated with early-onset inflammatory bowel disease and found that employing hematopoietic cell transplantation (HCT) using treosulfan-based conditioning results in excellent survival with full donor engraftment. Importantly, anakinra effectively serves as a bridge to HCT.

Deficiency of IL-10 and IL-10R represents a group of inborn errors of immunity associated with monogenic very early-onset inflammatory bowel disease (1). Patients typically present in infancy with chronic diarrhea, bloody diarrhea, or colitis. Additional features include perianal fistulae, abscesses and fissures, and failure to thrive. Extra-intestinal findings such as folliculitis, oral ulcers, and arthritis have been reported. Historically, immunosuppressive therapies including steroids, azathioprine, cyclosporine, and infliximab have been used to suppress/control the immune dysregulation but with limited success (2). Newer therapies, such as anakinra, a recombinant IL-1 receptor antagonist, have shown promise in controlling the disease but are not curative (3). Hematopoietic cell transplantation (HCT) can be curative and lead to remission of inflammatory bowel disease (IBD) in affected patients (1, 2). We report two patients who underwent HCT using treosulfan-based conditioning for treatment of IL-10RA deficiency. Treosulfan is a prodrug of an alkylating agent structurally related to busulfan. It has both myeloablative and immunosuppressive properties and has increasingly been used in HCT for both malignant and nonmalignant diseases with low transplant-related mortality and toxicity (4). The two cases presented here span nearly a decade in time, revealing the long-term efficacy of a similar HCT approach. Furthermore, this report also validates novel biallelic variants in *IL10RA* and highlights the more recent use of agents such as anakinra as a bridge to HCT. Written and informed consent was obtained for all participants, and studies were approved by the institutional review board (RG1005072, RG2809001).

The first patient is a now 24-year-old non-Hispanic black male born to consanguineous parents who initially presented at 9 days of age with a diffuse pustular rash. Around 1 mo of age, he developed bloody stools followed by rectal prolapse, and by 3 mo of age, he was found to have severe fistulizing colitis and underwent a diverting ileostomy. His course was further complicated by infections including recurrent pneumonia and intra-abdominal abscesses, significant aspiration in the setting of gastroesophageal reflux requiring Nissen fundoplication, feeding difficulties requiring i.v. and later enteral (via gastrostomy) nutritional support, and sensorineural hearing loss. There was no sustained meaningful response despite trying multiple immunosuppressive therapies including steroids, azathioprine, 6-mercaptopurine, infliximab, adalimumab, and methotrexate. Due to concerns for an underlying immune defect, he ultimately underwent genetic testing that demonstrated novel homozygous pathogenic missense variants in *IL10RA* located in the extracellular domain of the protein (c.506T>C, p.Ile169Thr) at position chr11:117,993,379 T>C (GRCh38) with a Combined Annotation-Dependent Depletion (CADD) (v1.7) score of 23.6. Although the variants had not yet been reported at the time of diagnosis, the genetic findings were consistent with the clinical phenotype, and the variants have since been published (5). Prior to HCT, additional testing was performed that demonstrated abnormal IL-10 signaling with no significant STAT3 phosphorylation in response to IL-10 stimulation, consistent with IL-10 receptor deficiency.

**Figure 1.**
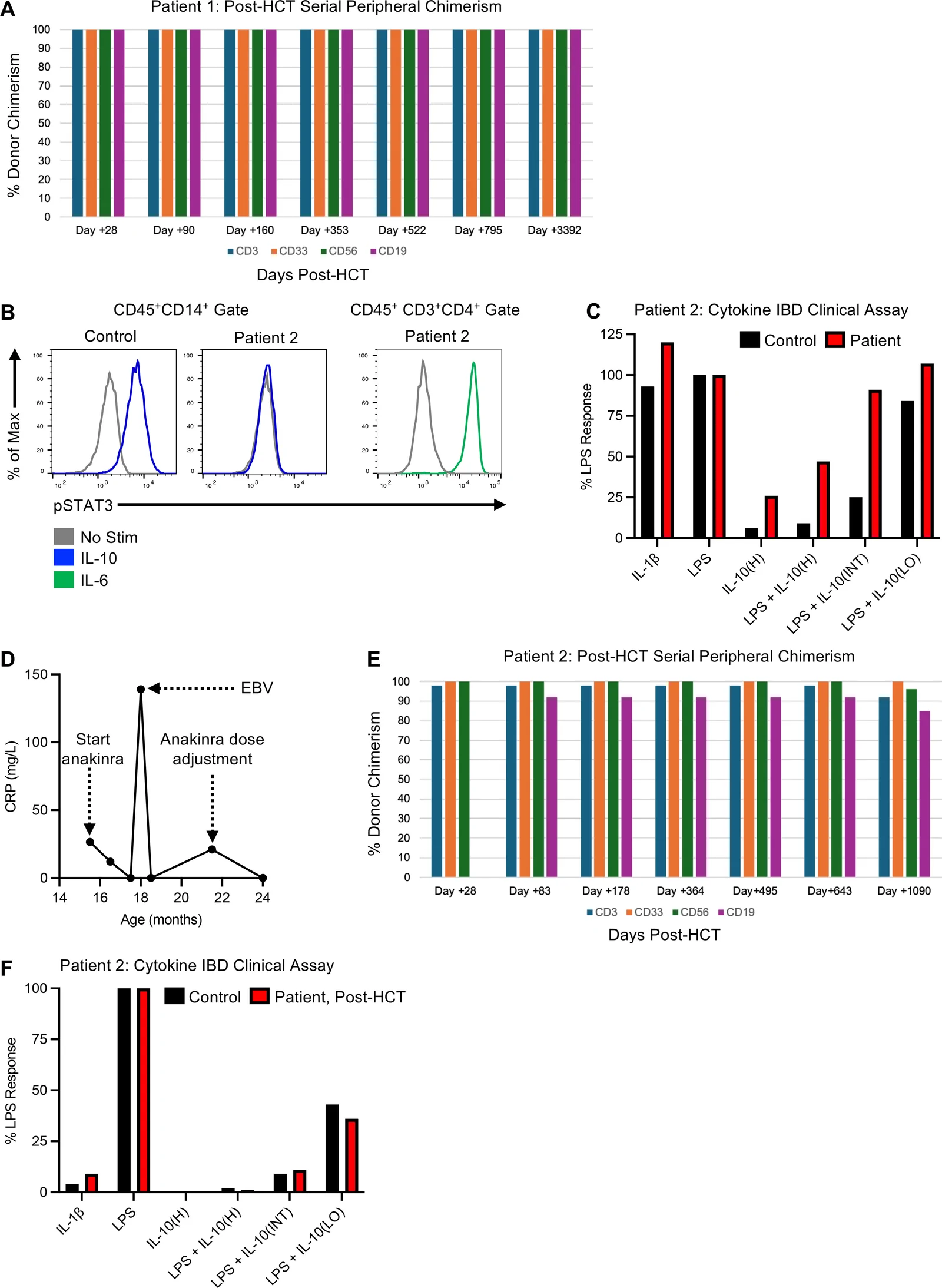
**Clinical, genetic, and functional analysis of *IL10RA* variants. (A)** Serial post-HCT peripheral blood donor chimerism shown for patient 1. **(B)** Patient 2 and control PBMCs were stimulated with IL-10 (50 ng/ml, PeproTech) for 30 min, and pSTAT3 (pY705, Cat# 557815; BioLegend) was assessed by flow cytometry shown as unstimulated (gray) vs. stimulated (blue) gated on live CD45^+^CD14^+^ cells. On the right panel, pSTAT3 staining is shown, comparing unstimulated (gray) vs. IL-6 (10 mg/ml, Miltenyi) stimulated (green) PBMCs from the patient, gated on live CD45^+^CD3^+^CD4^+^ cells. Data are representative of two independent assays. **(C)** Graphical representation of clinical testing for IL-10 responsiveness in patient PBMCs (patient 2) compared with the control at diagnosis. Cells were incubated with the indicated stimulants with or without IL-10 (H, high; INT, intermediate; and LO, low concentration) and IL-6 production as a percentage of the positive control (LPS) is graphed on the y axis. The assay is representative of at least three clinical assays obtained. **(D)** CRP response to anakinra before HCT in patient 2. **(E)** Serial post-HCT peripheral blood donor chimerism shown for patient 2. **(F)** Graphical representation of clinical testing for IL-10 responsiveness in patient PBMCs (patient 2) compared with the control, 1 year after HCT.

At 11.6 years of age, he underwent an HLA-mismatched (4/6 or 6/8) cord blood HCT following conditioning with treosulfan (total dose 42 g/m^2^; day −6 to day −4), fludarabine (total dose 150 mg/m^2^; day −6 to day −2), rabbit anti-thymocyte globulin (rATG, total dose 6 mg/kg; day −4 to day −2), and total body irradiation (TBI) 300 cGY (day −1). Mycophenolate mofetil (MMF) and cyclosporine were given for graft-versus-host disease (GVHD) prophylaxis. He is currently 12 years post-HCT with 100% donor CD3 and CD33 engraftment at last evaluation (Fig. 1 A) and had normal STAT3 phosphorylation in lymphocytes stimulated with IL-10 and IL-21 at 1 year post-HCT (clinical assay). He did not develop acute or chronic GVHD.

Post-HCT complications included CMV reactivation at day +49 post-HCT that was successfully treated with ganciclovir. After his transplant, follow-up endoscopic exam showed dramatic healing of his prior severe anal ulceration/fissures and essentially no demonstrable inflammation. However, he remained with a rectal–anal junction stricture as a consequence of the long-standing severe inflammation predating the transplant. His last surgical procedure was a loop sigmoid colostomy ∼5 years after HCT with the prior ileostomy closed. Immune reconstitution has been good with vaccine response noted after HCT, and he has had no major infections.

The second patient is a now 6-year-old non-Hispanic white female who initially presented at 2 mo of age with a labial abscess that failed to improve with antibiotic therapy. She subsequently developed an anal fistula that was surgically repaired at 4 mo of age. At 8 mo of age, she was found to have a rectovaginal fistula and continued perirectal fistula, both of which were surgically repaired. Other clinical history was notable for three episodes of acute otitis media, croup, and pneumonia, and intermittent papular rashes treated as candidiasis. Due to concerns for an underlying immune deficiency, the patient underwent an immune evaluation, which included normal immunoglobulin levels, lymphocyte subset enumeration, and neutrophil oxidative burst. No histopathologic abnormalities were seen on gastrointestinal biopsies. An Invitae genetic panel (407 genes) testing for known inborn errors of immunity was obtained, and biallelic inherited variants of uncertain significance in *IL10RA* were detected. The first variant is a missense change located in the extracellular domain of the protein (c.416T>A, p.Ile139Asn) at position chr11:117,993,289 T>A (GRCh38) and is absent from gnomAD (v4.0.0) with a CADD (v1.7) score of 23.7. The second variant is predicted to lead to an early truncation (c.1495C>T, p.Gln499*) at position chr11:117,999,399 C>T (GRCh38) located in the cytosolic domain and is absent from gnomAD (v4.0.0) with a CADD (v1.7) score of 39. Based on her clinical phenotype and the predicted pathogenicity, there was high concern that the variants were associated with her disease. On a research basis, patient peripheral blood mononuclear cells (PBMCs) demonstrated a lack of response to IL-10 stimulation, as indicated by a failure to upregulate phosphorylated STAT3 (Fig. 1 B). pSTAT3 expression was normal in the patient PBMCs after stimulation with IL-6, suggesting that the defect was isolated to the IL-10 pathway (Fig. 1 B). Additional clinical testing (Medical College of Wisconsin Clinical Immunodiagnostic Laboratory, cytokine-IBD test) demonstrated that treatment of PBMCs with IL-10 failed to inhibit LPS-induced IL-6 production, confirming a functional defect of IL-10RA (Fig. 1 C). Interestingly, there was suppression observed at the highest dose of IL-10, which could be consistent with a hypomorphic variant.

The patient was referred for HCT evaluation based on her history, genetic variants, and functional testing. It was felt that control of her inflammation prior to HCT would lead to the best chance of success for future HCT. Based on her disease, elevated C-reactive protein (CRP), the known role of IL-10 in regulating IL-1 production in vitro and in vivo, and a prior report of anakinra use with IL-10RA deficiency (3), she was started on daily anakinra, 2 mg/kg subcutaneous injection. In humans, IL-10R signaling regulates inflammasome-mediated IL-1β production. Studies have shown that an inability to signal through the IL-10 receptor contributes to excessive IL-1β secretion and mucosal inflammation (3). Therapy directed against IL-1 has been shown effective in two patients with IL-10RA deficiency and served as a bridge to successful HCT in one patient (3). Her CRP normalized while on anakinra, energy improved, new gains in developmental milestones were observed, and she improved from the 15th to the 50th percentile for growth (weight) in the first 6 mo of treatment. CRP was elevated during an acute EBV infection and spontaneously resolved. Approximately 6 mo after anakinra, she had decreased energy, intermittent fevers, and elevated CRP. Anakinra dosage was weight-adjusted, fevers resolved, energy improved, and CRP was again undetectable (Fig. 1 D).

She was managed on anakinra as a bridge to definitive therapy with HCT, which she received at the age of 3 years. She underwent an HLA-matched (10/10) unrelated bone marrow HCT following conditioning with thiotepa (total dose 10 mg/kg; day −7), treosulfan (total dose 36 gm/m^2^; days −6 to −4), fludarabine (total dose 150 mg/m^2^/dose; days −6 to day −2), and rATG (total dose 6 mg/kg; days −4 to −2). GVHD prophylaxis included methotrexate and tacrolimus. She is currently 3 years post-HCT with donor chimerisms of 100% CD33^+^, 92% CD3^+^, 85% CD19^+^, and 96% CD56^+^ (Fig. 1 E). Repeat functional testing via the cytokine-IBD panel at 1 year post-HCT was normal (Fig. 1 F). She was diagnosed with acute grade II skin and gut GVHD (S3, G2, L0) at day +15 post-HCT, followed by delayed acute skin GVHD at day +188 post-HCT. Additional post-HCT complications included EBV viremia (peak EBV PCR: 1,410 IU/ml; day +64) that resolved after 3 doses of rituximab. Roughly 16 mo after HCT, she developed autoimmune hemolytic anemia and was treated with MMF and prednisone. She has successfully tapered off prednisone and remains on MMF, which is being weaned currently with improvement. She remains on subcutaneous immunoglobulin therapy (Hizentra) to maintain an IgG level of >600 mg/dl while on immune suppression. She has a persistent perianal fistula without communication and without signs of active inflammation or enteropathy. The fistula will be surgically reconstructed when off immune suppression.

In summary, treosulfan-based conditioning was successful in establishing full donor/high-level mixed engraftment following HCT with resolution of disease in patients with IL-10R deficiency. This report also characterizes new *IL10RA* variants and highlights the efficacy of IL-1 blockade as a bridging therapy to transplantation for autoinflammation associated with IL-10 signaling defects.

## Data Availability

The data underlying Fig. 1 are available in the published article and from the corresponding author upon request. Accession numbers from ClinVar: Homozygous variant for patient 1: NM_001558.4(IL-10RA):c.506T>C (p.Ile169Thr); Variation ID: 943905; Accession: VCV000943905.9. Variants for patient 2: first variant: NM_001558.4(IL-10RA):c.416T>A (p.Ile139Asn); Variation ID: 1490173; Accession: VCV001490173.8; second variant: NM_001558.4(IL-10RA):c.1495C>T (p.Gln499Ter); Variation ID: 1484994; Accession: VCV001484994.6.
